# Items

**Published:** 1904-10

**Authors:** 


### ITEMS.

### THE SUBSCRIBER AND THE LAW.

Following are some United States court decisions relative to subscriptions to periodicals:

1. Subscribers who do not give express notice to the contrary are considered as wishing to renew their subscriptions.

2. If subscribers order the discontinuance of their periodicals, the publisher may continue to send them until all arrearages are paid.

3. If subscribers neglect or refuse to take their periodicals from the postoffice to which they are directed, they are responsible until they have settled their bills and ordered them discontinued.

4. If subscribers move to other places without informing the publishers, and the papers are sent to the former address, they are held responsible.

5. The courts have decided that refusing to take periodicals from the office, or removing and leaving them uncalled for, is prima facie evidence of intentional fraud.

6. If subscribers pay in advance they are bound to give notice at the end of the time if they do not wish to continue taking it; otherwise the publisher is authorised to send it and the subscriber will be responsible until an express notice, with payment of all arrearages, is sent to the publisher.

The latest postal laws are such that newspaper publishers can arrest anyone for fraud who takes a paper and refuses to pay for it. Under this law the man who allows his subscription to run for some time unpaid, and then orders it discontinued, or orders the postmaster to mark it "refused" and have a postal card sent notifying the publisher, leaves himself liable to arrest and fine the same as for theft.—*Atlanta Medical and Surgical Journal.*

---

Now is the time for the World's Fair. Weather and traveling at their best. Tickets by the Wabash and live at The Monticello, St. Louis.

